# Towards a Knowledge-Based Decision Support System for Integrated Control of Woolly Apple Aphid, *Eriosoma lanigerum*, with Maximal Biological Suppression by the Parasitoid *Aphelinus mali*

**DOI:** 10.3390/insects12060479

**Published:** 2021-05-21

**Authors:** Eva Bangels, Ammar Alhmedi, Wannes Akkermans, Dany Bylemans, Tim Belien

**Affiliations:** 1 Zoology Department, Research Centre for Fruit Cultivation (Pcfruit Npo), Fruittuinweg 1, B-3800 Sint-Truiden, Belgium; eva.bangels@pcfruit.be (E.B.); ammar.alhmedi@pcfruit.be (A.A.); wannes.akkermans@outlook.com (W.A.); dany.bylemans@pcfruit.be (D.B.); 2 Department of Biosystems, KU Leuven, Decroylaan 42, B-3001 Heverlee, Belgium

**Keywords:** woolly apple aphid *Eriosoma lanigerum*, parasitoid *Apheninus mali*, population dynamics, decision support

## Abstract

**Simple Summary:**

The woolly apple aphid *Eriosoma lanigerum* is an important pest in apple orchards worldwide. At present, effective limitation of woolly aphid populations relies on a good synergy between chemical control treatments and biological suppression by beneficial insects, especially by its main specific natural enemy, the parasitic wasp (parasitoid) *Aphelinus mali*. In order to reach maximum control levels on woolly apple aphids and avoid negative side effects on *A. mali*, decision support for the optimal timing and positioning of control treatments is needed. In this study, we developed prediction models that based on the weather conditions (temperature data) can reasonably accurately predict crucial development/activity phases of both insects in the orchard. These prediction models can be utilized to target insecticide sprayings at the most sensitive stage of the pest (woolly apple aphids) and/or to avoid insecticide sprayings with detrimental side effects at the vulnerable stage of the beneficial insect (parasitoid *A. mali*), as was demonstrated by the outcomes of a field trial in this study.

**Abstract:**

The woolly apple aphid *Eriosoma lanigerum* (Homoptera: Aphidiae) is an important pest in apple orchards worldwide. Since the withdrawal or restricted use of certain broad-spectrum insecticides, *E. lanigerum* has become one of the most severe pests in apple growing areas across Western Europe. At present, effective limitation of woolly aphid populations relies on a good synergy between chemical control treatments and biological suppression by beneficial arthropods, especially by its main specific natural enemy, the parasitoid *Aphelinus mali* (Hymenoptera: Aphelinidae). To develop a knowledge-based decision support system, detailed monitoring data of both species were collected in the field (region of Sint-Truiden, Belgium) for a period of ten years (2010–2020). *Aphelinus mali* flights were monitored in the field, starting before flowering until the end of the second-generation flight at minimum. The seasonal occurrence of the most important management stages of *E. lanigerum*, e.g., start of wool production or activity on aerial parts in spring and migration of crawlers from colonies towards flower clusters or shoots, were thoroughly monitored. All obtained data were compared with historical and literature data and analysed in a population dynamics phenological model. Our outcomes showed that the emergence of first-generation *A. mali* adults (critical for the first parasitation activity and the basis for following *A. mali* generations in the continuation of the season) can be accurately predicted by the developed model. Hence, this information can be utilized to avoid insecticide sprayings with detrimental side effects at this particular moment as demonstrated by the outcomes of a field trial. In addition, the start of migration of *E. lanigerum* crawlers towards flower clusters or shoots is accurately predicted by the model. In conclusion, our results demonstrate that the model can be used as decision support system for the optimal timing of control treatments in order to achieve effective control of *E. lanigerum* with maximal biological suppression by its main natural enemy.

## 1. Introduction

*Eriosoma lanigerum* (Hausmann) is one of the most detrimental pests in apple orchards [1,2,3,4,5]. It weakens the apple tree by feeding on bark, branches, shoots and roots, often resulting in gall-like swellings. Over and above the weakening of the apple trees, infestations with woolly apple aphids lead to production of smaller-sized fruits. The aphids produce long cottony-white wax filaments, providing a protective coat to the colonies and giving them a distinctive woolly appearance. In addition to colonisation of the aerial parts of the tree by migration of nymphs (“crawlers”), woolly apple aphids may be found year-round on roots of mature trees where they often go unnoticed [6]. Both features (woolly protective layer and below soil level hidden life forms) make them particularly difficult to control. In Western Europe, nearly only apterous virginoparae are of importance in commercial apple orchards [7], and they reach up to 12 generations/year [8]. Since the overwintering host, American elm (*Ulmus americana* L.) is not present in the Belgian pome fruit production regions, *E. lanigerum* completes its full life cycle on apple trees. Consequently, only rarely winged *E. lanigerum* morphs and eggs are spotted (observations by pcfruit on 28 September 2018, 22 September 2017 and 7 October 2014, Kerkom, Sint-Truiden). The potential dispersal role of winged woolly apple aphids, that are considered to produce sexual forms whose progeny can only grow on American elm [9], remains unclear in our region. Although overwintering occurs mainly as first instar nymphs [7], all instars as well as adults are observed by pcfruit during winter in the monitored orchards around Sint-Truiden.

Since 1961, monitoring data were collected at pcfruit for the Sint-Truiden region. Initially, monitoring was concentrated on the start of waxy filament growth on the aphids (thus, when the naked (without wax covering) aphids become active again and start to feed, moult and produce the so-called wool). Though, since the widely adoption of integrated pest management (IPM) tactics, with the transition from broad-spectrum insecticides to more selective crop protection products, the migration of crawlers has become the main target in woolly apple aphid control. Consequently, in the last decades, the focus of monitoring activities was shifted to this life stage.

Besides, a list of predators that feed on woolly apple aphids, such as syrphids, coccinellids, chrysopids, carabids, spiders and earwigs [2,10,11,12,13,14,15], one of the most important natural enemies of *E. lanigerum* worldwide is the parasitoid *Aphelinus mali* (Haldeman 1851) (Hymenoptera: Aphelinidae) [16,17,18]. This host-specific (solitary) endoparasitoid parasitizes all parthenogenetic stages of woolly apple aphids with a preference for third stage nymphs and older hosts, but has the disadvantage of having fewer generations per year (4–7 generations per year) compared to its host (10–12 generations per year) [3,19]. Moreover, unlike its host, *A. mali* is highly susceptible to insecticide applications, especially in the adult life-stage [3,5,8,20,21]. *A. mali* hibernates as a full-grown larva or pupa inside a dead hardened (mummified) body of a woolly apple aphid, and new adult parasitoid wasps emerge in spring [3,7,17].

Development times of all life stages of apterous virginoparae of *E. lanigerum* woolly apple aphids at constant temperatures of 10, 13, 15, 20, 25, 30 and 32 °C were reported by Asante [22]. A good linear model fit between developmental rate and temperature was observed for all life stages in the range 10–25 °C. The lower and upper temperature thresholds for total development were estimated at 5.2 and 32 °C, respectively. Mean degree-day accumulations required for completion of first, second, third and fourth instars and total development were: 125.6, 51.0, 47.7, 50.7 and 267.6, respectively [22].

*A. mali* development is also inversely based on temperature. Asante & Danthanarayana (1992) observed a good linear model fit between developmental rate and temperature in the range from 13 to 30 °C. Based on their findings, the use of a single linear regression model using 8.3 °C as a common lower threshold value for all life stages is sufficiently accurate to predict the seasonal development of this parasitoid. With this notional developmental threshold of 8.3 °C, a need of 254.8 degree-days (DD) above the lower threshold was presented to complete development from time of oviposition to adult emergence for both sexes combined [17].

Temperature thresholds and DD temperature sums, with a total development time for apterous virginoparae woolly apple aphids after 267.6 DD with a minimum threshold of 5.2 °C [22] versus a minimum threshold of 8.3 °C and 254.8 DD from egg to adult for *A. mali* confirm a head start or continuous lead for *E. lanigerum* versus its parasitoid *A. mali*. In order to reach maximum control levels on woolly apple aphids with maximal biological suppression by *A. mali*, decision support for the optimal timing and positioning of control treatments is needed. The goal of this study was to build phenology prediction models based on the literature and observational data for *A. mali* as well as *E. lanigerum*, and validate their usability for decision making in integrated crop protection in apple growing practice. More specifically, we aimed to determine the accuracy of prediction of the first- and second-generation flights of *A. mali* as well as the onset of migration of the first *E. lanigerum* crawlers. After all, these are crucial moments for the application of crop protection sprays against woolly apple aphids where negative impact on their main natural enemy (*A. mali*) must be absolutely avoided. This research is a continuation of our research reported in 2011 [3,5].

## 2. Materials and Methods

### 2.1. Monitoring

During 11 subsequent years, the *Aphelinus mali* flight was assessed from April (phenology stage of green to pink bud) to minimum July, at least weekly (3–7 days interval), quantifying the number of adults on 3 yellow sticky traps type Bug-Scan^®^ Yellow (Biobest, Westerlo, Belgium) 25 × 10 cm² per monitored orchard [23]. Traps were placed at about 1.5 m height in the canopy of the apple tree. Since 2010, monitoring was executed in IPM apple orchards (Jonagold mutants, Greenstar or Braeburn varieties) in or within a maximum radius of 10 km around the pcfruit research centre located in the east of Belgium) (50°46′22.05″ N, 5°9′37.51″ E). In apple orchards (2–5 depending on the monitoring year) within the same radius around Sint-Truiden, at the end of winter and during (early) spring, *E. lanigerum* phenology was monitored, especially the start of wool formation on the overwintering population (on old wounds on the trunk) and crawler movement or migration of nymphs towards the flower clusters or shoots (=the first date that occurrence of *E. lanigerum* nymphs on these structures is observed was registered every monitoring year). In every monitored orchard, at least 10 of the above-mentioned plant structures (old woods on the trunk, flower clusters, shoots) were carefully inspected in each monitoring year of this study.

### 2.2. Modelling

*E. lanigerum* development times required for completion of the four instars and total development as well as the *A. mali* egg-to-adult development time at different constant temperatures are available in the literature for 10 °C (only *E. lanigerum*), 13, 15, 18, 20, 25 and 30 °C [17,22]. For both species, a temperature-driven phenological model was built based on a saturated polynomial regression. The development rates (=1/development time (days)) were fitted as a function of temperature (x = T (°C), f(x) = 1/development time (days) = development rate) using the R studio software (version R 3.6.2) and the “devRate” R package [24]. For this purpose, the “devRate” R package functions and datasets/lists which include polynomial 2 (“poly2”) and polynomial 4 (“poly4”) functions were extended with the formulas for the polynomial 5 and polynomial 6 functions (see Supplementary Table S1). The “devRate” R package uses a nonlinear least-squares estimation procedure for parameter estimation. Models’ adequation was evaluated through diagnostic residual plots and the Akaike Information Criterion (AIC) (see Supplementary Table S1). The plots of the development rates as a function of constant temperatures are shown in Supplementary Figures S1 and S2. The development times and generations with respect to environmental temperatures were estimated using the “devRateIBM” function of the “devRate” R-package, with the assumptions that *A. mali* females immediately mate and lay eggs after emergence (based on earlier observations [25]), and apterous virginoparae woolly apple aphids immediately give birth to new nymphs after becoming adult (based on earlier observations [22]). We simulated 50 individuals, with a stochasticity in development rate centred on the development rate, with a standard deviation of 0.015 (normal distribution). The predicted first dates of the first- and second-generation (G1 and G2) *A. mali* adults and the predicted first dates of the new first instar virginoparae *E. lanigerum* “crawlers” based on daily temperature data from 2010–2020 were compared to data of field observations for each year of this period. For threshold temperatures below a minimum of 5.2 °C and 8.3 °C for *E. lanigerum* and *A. mali*, respectively, the development rates were set to zero (also at higher temperatures (above 32.7 °C) when the polynomial functions produce a theoretically negative (but physically impossible) negative development rate).

In order to quantify the accuracy of the predictions, the performance of the prediction models was assessed by examining agreement between observed and predicted dates (number of dates after 31/03) using linear regressions of observed (y) on predicted (x) dates [26] for the 11 years (2010–2020), with detailed monitoring and weather data. Observational monitoring data were collected in the area of Sint-Truiden, Belgium (as described in Section 2.1). For the same area, temperature data originating from Metinet (a network of automatic weather stations for agro-meteorological data in Flanders), located inside apple or pear orchards, were included. An ideal model would be both precise and accurate, as evidenced by a correlation coefficient r = 1, and a least squares regression line of y = x [27]. All statistical analyses were performed with the Rstudio software (version R 3.6.2).

### 2.3. Field Study in Apple Orchard

A field trial was carried out in 2020 in an apple orchard (variety Boskoop, located at 50°47’24.60″ N 5°16’04.60″ E) with a homogenous presence of *E. lanigerum* as well as *A. mali*, following the guidelines as described in EPPO (European and Mediterranean Plant Protection Organization) standard PP1/254(1) (*E. lanigerum*) and the recommendations for evaluating the effects on non-target arthropods (*A. mali* versus *E. lanigerum*) [28,29]. The trial was set up in a fully randomised block design, with 4 replicates, including the untreated control and a treatment with Decis 15 EW (containing 15 g/L deltamethrin). These treatments were part of a bigger trial, with other treatments which are not reported in this article, but which were (statistically) analysed together. Each trial plot consisted of 7 trees (3.10 m treated tree height with a plant distance within the row of 1.75 m), which were sprayed using a motorized backpack sprayer (Type Stihl, model SR 430, Stihl, Puurs-Sint-Amands, Belgium) with a 45° deflector screen on the output tube to be able to reach the woolly apple aphids (and *A. mali* adults) at the underside of the branches. Decis 15 EW was sprayed on 16 April 2020 with a dose rate of 0.300 L/ha Leaf Wall Area (LWA) (=4.5 g deltamethrin/ha LWA) using a water volume of 333.3 L/ha LWA. Since apple trees are a vertical crop, the product dose rate and water volume are expressed per ha LWA, as recommended by EPPO (guideline PP1/239(3) [30]). The LWA is calculated by the number of trees × planting distance within the row (d) × treated tree height × 2 sides, and corresponded to 15,500 m^2^/ha soil surface in the trial orchard.

For each plot, the total woolly apple aphid colony surface (cm^2^) on 10 previously marked areas per plot of infested shoots (2 years old + new growth) was assessed using a mesh (with squares of 0.5–1 cm^2^) that was held against the colonies. In addition, the number of observed *A. mali* adults and, subsequently, the number of parasitized (mummified) woolly apple aphids (opened as well as closed) was assessed on the same areas (if dense wool was present, the wax was first removed by gently blowing, in order to be able to count the aphids/mummies with the naked eye). The degree of parasitism was calculated by the number of parasitized aphids per 10 cm^2^ *E. lanigerum* colony surface.

Trial results were statistically analysed using the Unistat Statistical Package, version 10 (Unistat Ltd., London, UK). After confirming the homogeneity of variances with Bartlett’s Chi-Square and Bartlett–Box F-test, a GLM (General Linear Model) procedure with different outputs options (Anova, Table of means, Plot of residuals, Multiple comparisons) was executed. Treatment means were separated by the Student–Newman–Keuls multiple range test (5% level).

## 3. Results

### 3.1. Polynomial Regression Models for A. mali and E. lanigerum

The following polynomial function represents the egg-to-adult development rate as a function of the temperature (°C) for *A. mali*:

f(x) = −2.940 × 10^−3^ − 1.716 × 10^−4^x + 4.895 × 10^−4^x^2^ − 5.389 × 10^−5^x^3^ + 2.653 × 10^−6^x^4^ − 4.303 × 10^−8^x^5^ (1)

The following polynomial function represents the development rate for apterous virginoparae *E. lanigerum* woolly apple aphids as a function of the temperature (°C):

f(x) = 1.013 × 10^−3^ − 9.014 × 10^−4^x − 2.011 × 10^−4^x^2^ + 1.044 × 10^−4^x^3^ − 8.425 × 10^−6^x^4^ + 2.731 × 10^−7^x^5^ − 3.209 × 10^−9^x^6^ (2)

**Figure 1:**
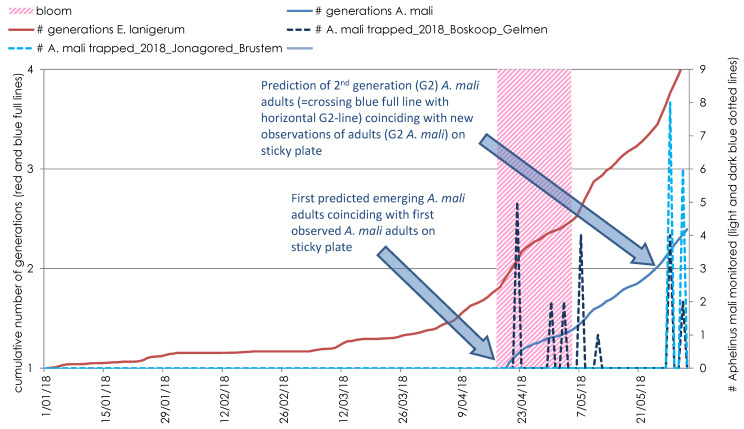
The development of *E. lanigerum* and *A. mali* as predicted (red and blue line, respectively) and as monitored *A. mali* adults at sticky plates (dotted lines) in the orchard at two different locations in the region of Sint-Truiden (apple cultivar Jonagored in Brustem and apple cultivar Boskoop in Gelmen) in 2018.

Based on the calculated polynomial regression models and actual orchard temperature data, the continuous development for both species was calculated and cumulatively summed (for temperatures below or above the threshold temperatures, no development was cumulatively added) (see Figure 1 for the output graph for 2018). Since *A. mali* mainly overwinters as a full-grown larva and *A. mali* eggs, larval and pupal development times relate to each other as 1:4:5 [7,17,31], it is assumed that all these overwintering forms were halfway through their development from egg to adult at the start of the year (01/01). So, the first new emerging *A. mali* adults (=start of the first *A. mali* flights in the season) in spring are predicted when the remaining half of the development time has been completed, which corresponds to 50% of the total egg-to-adult timespan (Figure 1). The appearance of the second-generation *A. mali* first instars is predicted when the next full generation time is reached. As *E. lanigerum* overwinters mainly as first instar nymphs [7], it was assumed that all woolly apple aphids are at the first nymphal stage at the start of the year (01/01). Since the new first instar virginoparae (“crawlers”) are regarded as the first migrating forms, dispersing from parent colonies to form new colonies in the tree canopy [31], the start of migration is predicted when *E. lanigerum* has completed one full generation time (Figure 1).

### 3.2. Accuracy of A. mali First and Second Generation Adults Emergence Predictions

The predicted development based on the measured hourly temperature data was compared with field monitoring data of *A. mali* adults from 2010 to 2020. In Table 1, the predicted and observed dates for the new first-generation (G1) *A. mali* adults (=start of the *A. mali* flights in the season) and second-generation (G2) *A. mali* adults are displayed.

**Figure 2:**
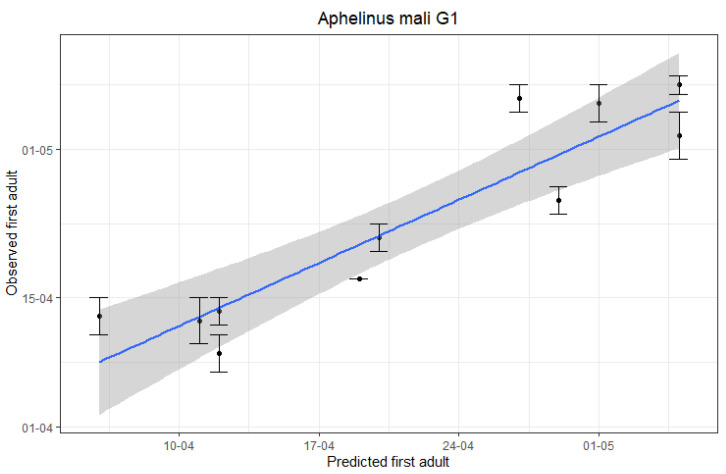
Linear regression analysis of predicted first-generation (G1) *A. mali* adults vs. observed first G1 *A. mali* adults by monitoring in apple orchards. Error bars represent the last date when yellow sticky monitoring plates were checked for which none of the checked plates showed caught *A. mali* adults and the first date that newly emerged *A. mali* adult(s) were detected on the monitoring plates.

**Figure 3:**
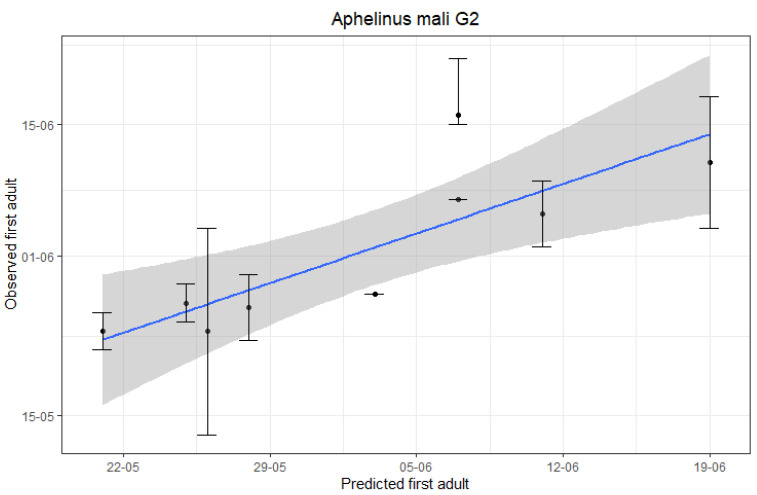
Linear regression analysis of predicted second-generation (G2) *A. mali* adults vs. observed second G2 *A. mali* adults by monitoring in apple orchards. Error bars represent the last date when yellow sticky monitoring plates were checked after the end of the G1 flights and for which none of the checked plates showed newly caught *A. mali* adults and the first date that newly emerging G2 *A. mali* adults were again detected on the sticky plates.

The accuracy of the predictions was assessed by linear regressions of observed (y) on predicted (x) dates. The outcomes are displayed in Figure 2. Our results indicate that the first-generation *A. mali* adult emergence can be predicted accurately based on hourly temperature data (R-squared = 0.8498, *p*-value < 0.001) with a mean error of ± 4 days. In addition, for the appearance of the new second-generation *A. mali* adults, a less accurate but still fairly good prediction was obtained (R-squared = 0.6897, *p*-value = 0.006) with a mean error of ±5 days (Figure 3).

### 3.3. Accuracy of E. lanigerum Migration Predictions

In addition, for woolly apple aphids, the predicted development based on the measured hourly temperature data was compared with field monitoring data. In particular, the moment of first migration of crawlers (new 1st instars of first *E. lanigerum* generation in the season) to flower clusters or new shoots was carefully registered via field monitoring. An overview of the predicted and observed dates for first migration activity is displayed in Table 2.

**Figure 4:**
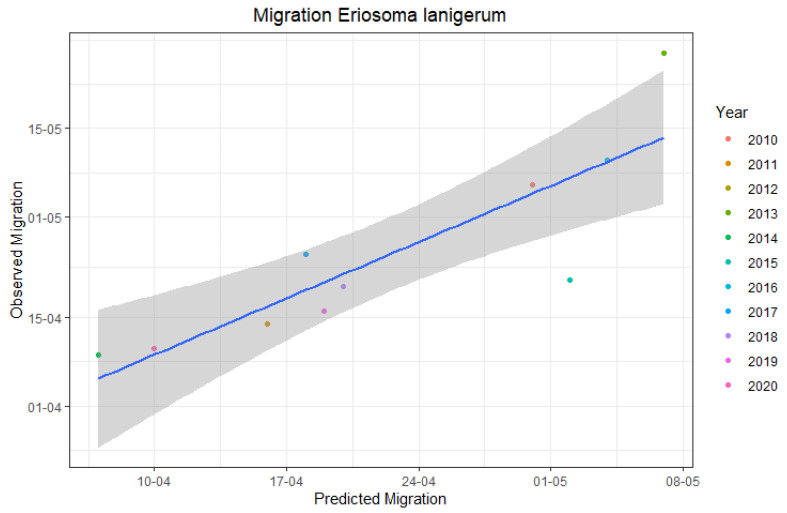
Linear regression analysis of predicted start of migration of *E. lanigerum* “crawlers” (=new first instar virginoparae) and observed dates of first migration activity in apple orchards monitored in this study.

The accuracy of the predictions was again assessed by linear regressions of observed (y) on predicted (x) dates (Figure 4). The outcomes indicate that the first migration of *E. lanigerum* crawlers can be predicted with a certain degree of accuracy based on hourly temperature data (R-squared = 0.7499, *p*-value = 0.001) with a mean error of ± 8 days.

### 3.4. Field Study on Side-Effects on A. mali and Impact on Biologicol Control of E. lanigerum

**Figure 5:**
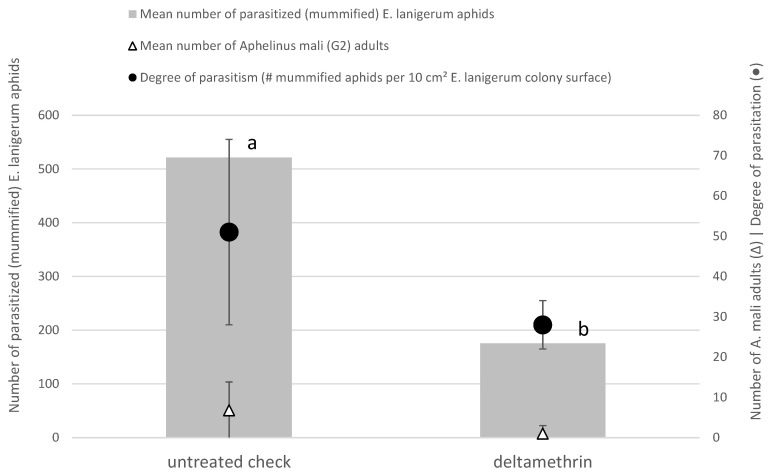
Results of field trial with deltamethrin sprayed at 4.5 g/ha LWA (Leaf Wall Area) on 16/04/2020 during the first flight period of *A. mali* adults. Assessments on 17/06/2020 of number of parasitized *E. lanigerum* aphids (grey bars, left vertical *Y*-axis), number of *A. mali* (G2) adults (with triangles) and total degree of parasitism (black circles) (both right vertical *Y*-axis) are shown. Statistical differences are indicated with different letters. Error bars represent the standard errors of counted numbers.

In the field trial, the broad-spectrum pyrethroid insecticide Decis 15 EW was sprayed at a dose rate of 4.5 g deltamethrin/ha LWA on 16 April 2020, during the first flight period of *A. mali* (G1) adults (see Table 1). Later on, the number of parasitized (mummified) aphids, and the appearance of new (G2) *A. mali* adults was assessed, and the degree of parasitism was determined. The outcomes for assessments on 16 April 2020 are shown in Figure 5. The insecticide spraying during the first flight period of *A. mali* (G1) adults resulted in a significant (*p* = 0.0243) decrease of the number of parasitized woolly apple aphids compared with the untreated check (mean numbers of 522 vs. 175 per assessed plot). This led to a 7-times smaller number of observed newly emerging G2 *A. mali* adults and a clear reduction of the parasitism degree (=mummified aphids per 10 cm^2^ *E. lanigerum* colony surface) (almost halving from a mean level of 51 ± 23 in the untreated check to 28 ± 6 in the deltamethrin-treated plots).

## 4. Discussion

In modern integrated and organic fruit production, parasitoids play an important role as main natural enemies of aphid pests [8,32]. Previous studies have indicated the importance of avoiding badly timed insecticide sprays on sensitive life stages of the parasitic wasp *A. mali*, allowing it to build its population to its full potential for the maximal natural suppression of woolly apple aphids in apple orchards [3,5,20,33,34]. Although yellow sticky plates are proven to be an efficient scouting method [35], this monitoring method is quite labour-intensive and far from obvious for implementation by fruit growers who are usually not entomologists. Therefore, there is a need for a prediction model that identifies the critical periods in the life cycle/dynamics of *A. mali*, and with that also the most optimal control opportunities of woolly apple aphids. In this study, we demonstrate that relatively simple degree-day phenology models derived from non-linear regression of known data on development rates of different life stages at constant temperatures [17,22] can reasonably accurately predict crucial development/activity phases of *A. mali* as well as *E. lanigerum* in the orchard. Undoubtedly, the most crucial stage, which makes the further built-up of a parasitoid population at all possible, are the first-generation *A. mali* adults emerging from their mummified woolly apple aphids in which they have overwintered. Firstly, only a very small fraction of the *A. mali* population successfully overwinters in these temperate climate conditions [7,19,22]. Secondly, these emerging adult parasitoid wasps are highly susceptible for pesticides [20,21,36]. Hence, the presented model can be used in extension services and warning systems to alert growers for the first-generation *A. mali* adult flight activity. This initial small flight activity peak usually has a short duration (typically 8–18 days) according to our monitoring data, which is in line with the observations of other studies in comparable climatological conditions [7,17,19,25,37].

In the field trial described in this study, the *A. mali* predictive model was used for a worst-case timing of a broad-spectrum insecticide, targeting the first generation of actively flying *A. mali* adults, leading to significant reduced biological suppression of *E. lanigerum* in the trial outcomes. In the crop protection practices in apple orchards, the model can be used to time insecticide sprays in a way that absolutely spares the first flight activity of (highly vulnerable) adult *A. mali* parasitoids and, preferably, also not during the appearance of second-generation *A. mali* flights. Hence, this is when the first rounds of parasitization have been successfully completed and the continuation and buildup of the parasitic wasps is guaranteed. Sprayings outside these sensitive time periods are still possible, as also confirmed by our earlier work in the development and testing of IPM complementary control strategies against *E. lanigerum*, which is also in line with studies of other authors [3,5,12,33]. As modern selective chemical or biological insecticides are usually most effective when sprayed against the (unprotected naked) crawlers when they are migrating to settle new colonies [33,38,39,40], the rather accurate prediction of the start of this migration activity is an interesting feature of our *E. lanigerum* model.

The here described models predict fairly well important development/activity phases of *A. mali* and *E. lanigerum* strains, occurring in our Belgian apple orchards in which the models were validated. The results (Table 1 and Table 2) show a discrepancy between predicted and observed dates of maximally a few days. Since the biological variation in the orchard in terms of the onset of migration of woolly apple aphids and appearance of second-generation *A. mali* adults is expected to be in the same order of magnitude, this margin of error is not expected to imply a risk of bad timings of crop protection sprayings with respect to these life stages. However, taking into account the typical very short flight activity of the first-generation *A. mali* adults (8–18 days) the model’s predictions for this crucial and extremely vulnerable life stage should be used with an additional safety margin of a few days, or only with additional in-field monitoring actions (which can be guided by the model outcomes). Whether the models are of the same accuracy in other regions of fruit production remains to be investigated, as other strains of the *A. mali* and *E. lanigerum* potentially differ in their development rates and temperature thresholds. For instance, other interesting *A. mali* strains have been described with lower temperature thresholds and hence lower effective accumulated temperature requirements for completing their life cycle [19,37]. Obviously, these *A. mali* strains can occur earlier in spring and may therefore provide better control of woolly apple aphids at a lower population level of the pest. It should be noted that also other abiotic and biotic factors such as, for instance, the fertilization program or the specific (potentially resistant) rootstock or apple cultivar can have a considerable influence on the performance and development of *E. lanigerum* [41,42,43], and therefore also on the performance and development of *A. mali*. Hence, in further refinements of the models, this should ideally be taken into account. We anticipate in future research to further elaborate the models by including such factors and adjusting model parameters via extensive model fitting approaches, optimizing the goodness of fit by minimizing the residuals between experimental field observation data and predictions [44,45,46]. This way, the precision and accuracy of predictions could be further improved, making it ultimately possible to locally predict the onset of emergence of first- and second-generation *A. mali* adult parasitic wasps and migration of *E. lanigerum* crawlers to the exact day.

## 5. Conclusions

The here described modelling approach generates a fairly accurate prediction of the first- and second-generation flights of *A. mali*, as well as the onset of migration of the first *E. lanigerum* crawlers. This enables apple growers to time insecticide treatments in a way that avoids detrimental side effects on the first flights of the very sensitive adult life stage of the natural enemy *A. mali*, and allows targeting of the most susceptible life stage of the pest *E. lanigerum* (i.e., the unprotected naked migrating woolly apple aphid nymphs).

## Figures and Tables

**Table 1:** Predicted and observed dates of new first-generation (G1) *A. mali* adults (=start of the *A. mali* flights in the season) and second-generation (G2) *A. mali* adults.

Year	Predicted First G1 Adults	Observed First G1 Adults ^1^	Predicted First G2 Adults	Observed First G2 Adults ^2^
2010	28/04	24/04	11/06	NA ^3^
2011	11/04	04/04	20/05	NA ^3^
2012	30/04	05/05	06/06	15/06
2013	04/05	07/05	18/06	10/06
2014	05/04	12/04	25/05	23/05
2015	26/04	05/05	10/06	04/06
2016	04/05	01/05	06/06	06/06
2017	11/04	08/04	27/05	25/05
2018	19/04	20/04	24/05	26/05
2019	18/04	16/04	02/06	27/05
2020	10/04	11/04	20/05	23/05

^1^ The mean date between the last date when yellow sticky monitoring plates were checked for which none of the checked plates showed caught *A. mali* adults and the first date that newly emerged *A. mali* adult(s) were detected on the monitoring plates. ^2^ The mean date between the last date when yellow sticky monitoring plates were checked after the end of the G1 flights and for which none of the checked plates showed newly caught *A. mali* adults and the first date that newly emerging G2 *A. mali* adults were again detected on the sticky plates. ^3^ No detailed monitoring data available to be able to distinguish between *A. mali* G1 and G2.

**Table 2:** Predicted start of migration of *E. lanigerum* “crawlers” (=new first instar virginoparae) and observed dates of first migration activity in apple orchards monitored in this study.

Year	Predicted First Migration	Observed First Migration
2010	30/04	06/05
2011	16/04	14/04
2012	29/04	NA ^1^
2013	07/05	27/05
2014	07/04	09/04
2015	02/05	21/04
2016	03/05	09/05
2017	18/04	25/04
2018	20/04	20/04
2019	19/04	16/04
2020	09/04	09/04

^1^ No detailed monitoring data available to be able to pinpoint precisely the first date of crawler migration in apple trees.

## Data Availability

The data presented in this study are available on request from the corresponding author.

## Supplementary Materials

The following are available online at https://www.mdpi.com/article/10.3390/insects12060479/s1, Table S1: Model functions, estimated parameters and the AIC values for each model, Figure S1: Fitting of the fifth order polynomial function to describe the temperature-dependent development rate of *A. mali*, Figure S2: Fitting of the sixth order polynomial function to describe the temperature-dependent development rate of *E. lanigerum*.
