# Supplementary material for: Towards a Knowledge-Based Decision Support System for Integrated Control of Woolly Apple Aphid, Eriosoma lanigerum, with Maximal Biological Suppression by the Parasitoid Aphelinus mali
Source: Insects. 2021 May 21;12(6):479. doi: 10.3390/insects12060479 (PMC8224070; doi:10.3390/insects12060479)
Supplement: Supplementary file 1 [file insects-12-00479-s001.zip › insects-1187478-supplementary.pdf]

Supplementary Materials: The following are available online at www.mdpi.com/xxx/s1

Table S1. Model functions, estimated parameters and the AIC values for each model

Target insect	Model characteristics	
A. mali		
	Model name	Polynomial 5
	Formula	rT ~ a0 + a1 * T + a2 * T^2 + a3 * T^3 + a4 * T^4 + a5 * T^5
	Estimated parameters	a0 = -2.940e-03 ± 1.506e-03
		a1 = -1.716e-04 ± 5.192e-04
		a2 = 4.895e-04 ± 1.540e-04
		a3 = -5.389e-05 ± 1.558e-05
		a4 = 2.653e-06 ± 5.831e-07
		a5 = -4.303e-08 ± 7.281e-09
	Akaike Information Criterion (AIC)	-194.74
E. lanigerum		
	Model name	Polynomial 6
	Formula	rT ~ a0 + a1 * T + a2 * T^2 + a3 * T^3 + a4 * T^4 + a5 * T^5 + a6 * T^6
	Estimated parameters	a0 = 1.013e-03 ± 1.132e-03
		a1 = -9.014e-04 ± 3.583e-04
		a2 = -2.011e-04 ± 1.486e-04
		a3 = 1.044e-04 ± 2.662e-05
		a4 = -8.425e-06 ± 1.819e-06
		a5 = 2.731e-07 ± 5.344e-08
		a6 = -3.209e-09 ± 5.673e-10
	Akaike Information Criterion (AIC)	-188.99

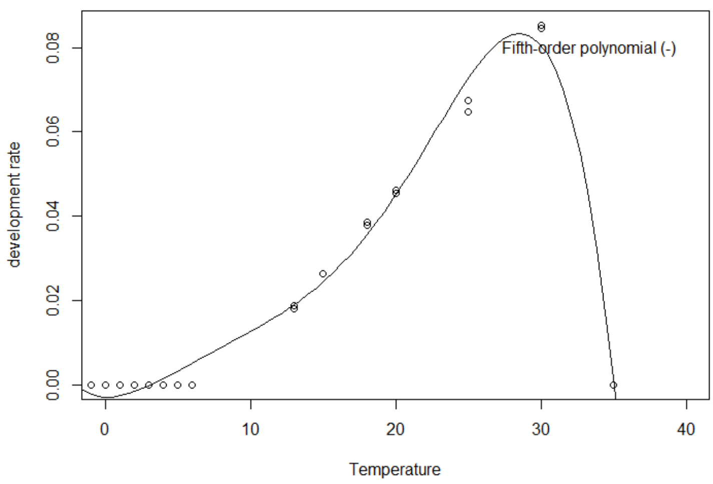

Figure S1. Fitting of the fifth order polynomial function to describe the temperature-dependent development rate of *A. mali*.

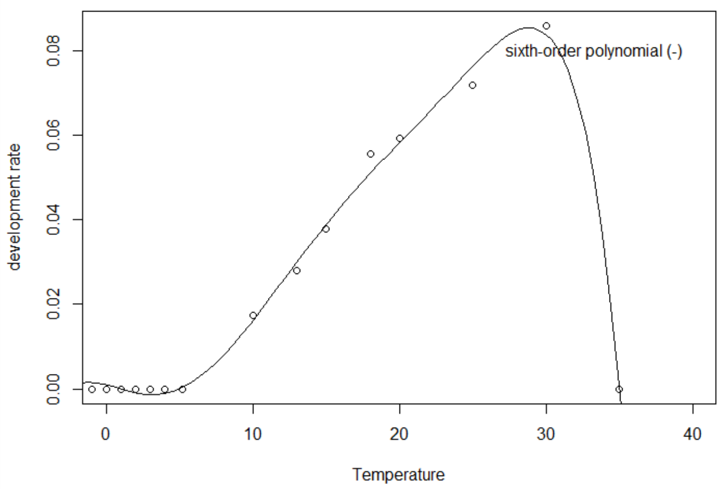

Figure S2. Fitting of the sixth order polynomial function to describe the temperature-dependent development rate of *E. lanigerum*.
